# Metformin represses cancer cells via alternate pathways in N-cadherin expressing vs. N-cadherin deficient cells

**DOI:** 10.18632/oncotarget.5023

**Published:** 2015-08-24

**Authors:** Rongbin Ge, Zongwei Wang, Shulin Wu, Yangjia Zhuo, Aleksandar G. Otsetov, Chao Cai, Weide Zhong, Chin-Lee Wu, Aria F. Olumi

**Affiliations:** ^1^ Department of Urology, Massachusetts General Hospital, Harvard Medical School, Boston, Massachusetts, USA; ^2^ Department of Pathology, Massachusetts General Hospital, Harvard Medical School, Boston, Massachusetts, USA; ^3^ Department of Urology, Guangdong Key Laboratory of Clinical Molecular Medicine and Diagnostics, Guangzhou First People's Hospital, Guangzhou Medical University, Guangzhou, China

**Keywords:** prostate cancer, metformin, TWIST, N-cadherin

## Abstract

Metformin has emerged as a potential anticancer agent. Here, we demonstrate that metformin plays an anti-tumor role via repressing N-cadherin, independent of AMPK, in wild-type N-cadherin cancer cells. Ectopic-expression of N-cadherin develops metformin-resistant cancer cells, while suppression of N-cadherin sensitizes cancer to metformin. Manipulation of AMPK expression does not alter sensitivity of cancer to metformin. We show that NF-kappaB is a downstream molecule of N-cadherin and metformin regulates NF-kappaB signaling via suppressing N-cadherin. Moreover, we also suggest that TWIST1 is an upstream molecule of N-cadherin/NF-kappaB signaling and manipulation of TWIST1 expression changes the sensitivity of cancer cells to metformin. In contrast to the cells that express N-cadherin, in N-cadherin deficient cells, metformin plays an anti-tumor role via activation of AMPK. Ectopic expression of N-cadherin makes cancer more resistant to metformin. Therefore, we suggest that metformin's anti-cancer therapeutic effect is mediated through different molecular mechanism in wild-type vs. deficient N-cadherin cancer cells. At last, we selected 49 out of 984 patients’ samples with prostatic cancer after radical prostatectomy (selection criteria: Gleason score ≥ 7 and all patients taking metformin) and showed levels of N-cadherin, p65 and AMPK could predict post-surgical recurrence in prostate cancer after treatment of metformin.

## INTRODUCTION

Prostate cancer is the most common cancer and second most common cause of cancer death in American men [1]. Diabetes is highly prevalent in the world and the latest report demonstrates that there are 29.1 million people (9.3%) of the population in the United States are affected by diabetes [2]. Commonly, patients with type II diabetes are treated with metformin, the first line insulin sensitizer. Interestingly, many recent epidemiological studies have shown that metformin, a biguanide hypoglycemic, not only lowers the glucose level, but also significantly reduces cancer incidence and improves cancer patients’ survival, including prostate cancer in diabetic men [3–5]. For instance, our recent study showed that metformin, but not other oral hypoglycemics, is associated with decreased risk of prostate cancer diagnosis [6]. Although it has been reported that the cancer-prevention function of metformin is associated with LKB1/AMPK [7, 8], mTOR [9], p53/REDD1 [9], pEGFR and IGF-1R [10], p27 and p21 [11], the more accurate underlying molecular mechanisms still need further evaluation.

In this study, we demonstrate, for the first time, that in wild-type N-cadherin cancer models, metformin plays an anti-tumor role through repressing TWIST1/N-cadherin/NF-kappaB signaling, independent of activation of AMPK. Up-regulation of TWIST1/N-cadherin/NF-kappaB pathway makes cancer more resistant to metformin. However, in N-cadherin deficient cancer cells, the anti-tumor's effects of metformin mainly depend on activation of AMPK. Down-regulation of AMPK makes cancer more resistant to metformin and over-expression of N-cadherin compromises AMPK-mediated pro-apoptotic activities. Finally, from our cohort of 984 patients who had undergone radical prostatectomy for management of prostate cancer, we identified 49 who were using Metformin for management of diabetes and were diagnosed to have Gleason 7 or greater prostate cancer on the final surgical pathology evaluation. We evaluated the prostate cancer samples for N-cadherin, p65 and AMPK expression and assessed whether these biomarkers may have any clinical utility.

## RESULTS

### Metformin inhibited proliferation of cancer cells via repressing N-cadherin

**Figure 1:**
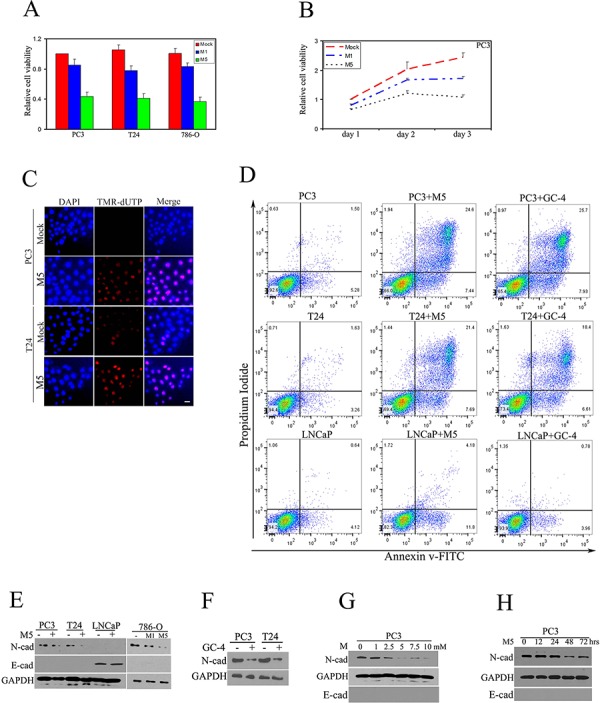
Metformin inhibited proliferation of cancer cells via repressing N-cadherin Prostate cancer cells (PC3), bladder cancer cells (T24) and renal cancer cells (786-0) were treated with metformin for 48 hours. **A.** MTS assay was performed to evaluate the cell viability. M1- 1 mM of metformin, M5- 5 mM of metformin. **B.** Metformin inhibited proliferation of PC3 cells in a time-dependent manner. **C.** Apoptotic TUNNEL assay was performed to detect cell death. **D.** Cells were treated as indicated, harvested, and apoptosis versus necrosis was quantified using AnnexinV-FITC and PI staining in FACS. Representative dot plots are displayed. (LNCaP: prostatic LNCaP cancer cells. GC-4: neutralizing N-cadherin antibody). **E.** Expression of N-cadherin, E-cadherin was detected by immunoblot. **F.** PC3 and T24 cells were treated with GC-4 (1:50) and N-cadherin was detected by immunoblot. Metformin-mediated inhibition of N-cadherin was dose-dependent. **G.** and time-dependent **H.** All experiments were repeated independently at least three times with similar results. (Scale bar, 100 μm)

To evaluate the anti-tumor effects of metformin, PC3 prostatic cancer cells, T24 bladder cancer cells and 786-O kidney cancer cells were treated with two different doses of metformin (1 mM, 5 mM), for 48 hours respectively. We found that 1 mM of metformin inhibited only 20% of cell viability and 5 mM of metformin inhibited 60–70% of cell viability in PC3 prostatic cancer cells. Similar results were observed in T24 bladder cancer cells and 786-O kidney cancer cells (Fig. 1A). To evaluate the optimal treatment duration, PC3 cells were treated with 1 mM or 5 mM of metformin for 24, 48, 72 hours respectively. The combination of 5 mM and 48 hours was considered an optimal experimental condition (Fig. 1B). Apoptotic activity, as measured by TUNEL assay and fluorescent activated cell sorting (FACS) showed increased cell death after treatment with metformin (5 mM, 48 hours) in PC3 and T24 cells (Fig. 1C). We also drew a comparison between metformin and N-cadherin neutralizing antibody GC-4 and found the effects of metformin-mediated apoptosis were comparable to effects of GC-4-mediated apoptosis in both cells (Fig. 1D).

Next, protein expression of N-cadherin and E-cadherin were measured in four types of prostate (PC3, LNCAP), bladder (T24), kidney cancer cells (786-O), we found that treatment of metformin (5 mM) dramatically repressed N-cadherin expression, but without altering E-cadherin expression levels. We also noted that there was no detectable N-cadherin level in LNCaP prostatic cancer cells (Fig. 1E). The expression of N-cadherin was reduced in GC-4 treated PC3 and T24 cancer cells (Fig. 1F). PC3 prostatic cancer cells treated with metformin showed a dose and time-dependent down-regulation of N-cadherin protein level (Fig. 1G–1H). These results suggest that metformin plays an anti-tumor role and represses N-cadherin expression. In Figure 1E, we observed LNCaP cells did not express N-cadherin, so we compared the sensitivity of PC3 (N-cadherin expressing) and LNCaP (N-cadherin decificient) cancer cells to metformin and found that LNCaP cells were sensitive to metformin, but resistant to N-cadherin neutralizing antibody GC-4 (Fig. 1D). These results suggest that metformin plays an antitumor role through different signaling pathways in N-cadherin expressing and N-cadherin deficient cells.

### N-cadherin regulates sensitivity of metformin-mediated apoptosis in N-cadherin expressing cancer cells, independent of AMPK

**Figure 2:**
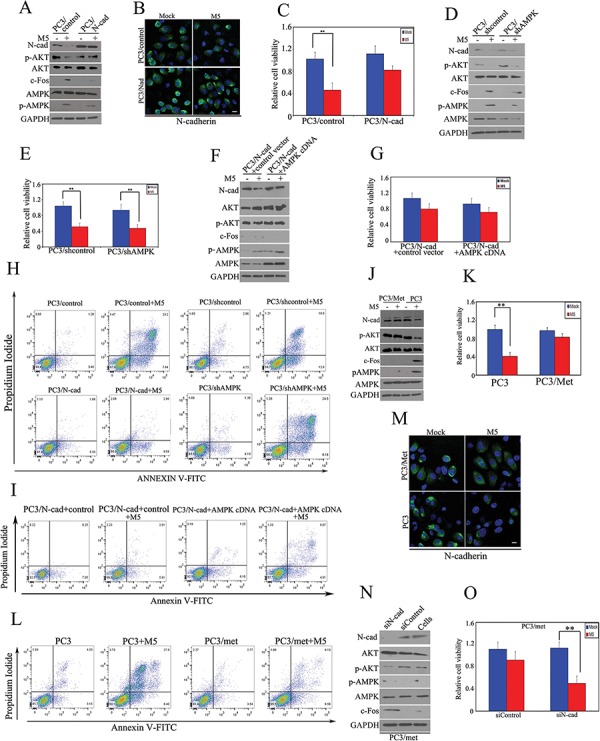
N-cadherin regulates sensitivity of metformin-mediated apoptosis in wild-type N-cadherin cancer cells, independently of AMPK **A.** PC3 cells were stably transfected with N-cadherin or control vectors, respectively and then the re-constructed stable PC3/N-cad cells were treated with 5 mM of metformin for 48 hours. N-cadherin, p-AKT, AKT, c-Fos, AMPK and p-AMPK were detected by immunoblot. **B.** Expression of N-cadherin was detected by immunofluoresence microscopy. **C.** Cell viability was evaluated by MTS assay. **D.** PC3 cells were stably transfected with shAMPK or control shRNA, and then the re-constructed stable PC3/shAMPK and PC3/control-shRNA cells were treated with 5 mM of metformin for 48 hours, respectively. Expression of N-cadherin, p-AKT, AKT, c-Fos and p-AMPK were detected by immunoblot. **E.** Cell viability was evaluated by MTS assay. **F.** AMPK or control vector was transiently transfected into PC3/N-cad stable cells. Expression of N-cadherin, p-AKT, AKT, c-Fos and p-AMPK were detected by immunoblot. **G.** Cell viability was evaluated by MTS assay. **H–I.** Apoptosis was detected by Annexin V assays. **J.** PC3/met stable cells were constructed as described in the methods section. PC3/met and PC3 cells were treated with 5 mM of metformin for 48 hours. Expression of N-cadherin, p-AKT, AKT, c-Fos and p-AMPK were detected by immunoblot. **K.** Cell viability was evaluated by MTS assay. **L.** Apoptosis was detected by Annexin V assays. **M.** Expression of N-cadherin was detected by immunofluoresence microscopy. **N.** N-cadherin siRNA was transfected into PC3/met. Expression of N-cadherin, AKT, p-AKT, p-AMPK and c-Fos was detected by immunoblot. **O.** Cell viability was evaluated by MTS assay. The data represent means of average determinants ± SEM. *P < 0.05; **P < 0.01. All experiments were repeated independently at least three times with similar results. (Scale bar, 100 μm)

To evaluate the importance of N-cadherin in regulating sensitivity to metformin, we stably over-expressed N-cadherin in PC3 cells and evaluated metformin-mediated antitumor activities. Down-regulation of N-cadherin and p-AKT, accompanied by a concomitant increase in c-Fos, was observed in PC3 cells after metformin treatment, consistent with signs of pro-apoptotic activity [12]. In contrast, over-expression of N-cadherin in PC3 cells compromised reactivation of c-Fos and significantly protected PC3 cells from metformin-mediated antitumor activities (Fig. 2A, 2C and 2H). We found that metformin readily inhibited N-cadherin expression in PC3/control cells, but not in the ectopically expressed N-cahderin in PC3/N-cad cells (Fig. 2B). In order to investigate if activation of AMPK plays a role in regulating sensitivity of metformin-mediated antitumor activities in cancer cells with N-cadherin expression, shAMPK was transfected into PC3 to generate a stable AMPK-knock-out PC3 cancer cells (PC3/shAMPK), and pAMPK expression was adequately suppressed (relative ratio of 1:0.48 in shcontrol vs. shAMPK – Fig. 2D). We found that metformin-mediated antitumor activities were not affected significantly after supressing AMPK (Fig. 2E and 2H). Next we over-expressed AMPK in PC3/N-cadherin stable cells (Fig. 2F) and found that over-expression of AMPK alone did not affect cell viability and apoptotic activities in metformin treated cells. Therefore, down-regulation of N-cadherin appeared to be necessary in metformin-mediated antitumor activity of cells expressing N-cadherin (Fig. 2G, 2I)

Moreover, PC3 cells were treated and selected with 5 mM of metformin for 10 weeks to generate a stable metformin-resistant PC3 cells (PC3/met cells). Down-regulated N-cadherin, p-AKT and concomitant upregulated c-Fos and p-AMPK were observed in wild type PC3 cells after long term metformin exposure. In contrast, protein expression of N-cadherin, p-AKT, c-Fos or p-AMPK was not affected by metformin in the resistant population of PC3/met cells (Fig. 2J), and they became more resistant to metformin (Fig. 2K-2L). We found that metformin readily inhibited N-cadherin expression in PC3/control cells, but not in the metformin-resistant PC3/met cells (Fig. 2M). Next, we suppressed N-cadherin in PC3/met cells using siN-cadherin and found that repression of N-cadherin sensitizes PC3/met to metformin-mediated antitumor activities (Fig. 2N–2O). In our previous study, we demonstrated that c-Fos, in addition to its proto-oncogenic activity, also possesses a pro-apoptotic function [12, 13]. The current study shows again that c-Fos might also play a pro-apoptotic role in metformin-mediated apoptosis. Moreover, these results show that N-cadherin, but not AMPK, mediates the antitumor effects of metformin in cancer cells that express N-cadherin.

### Pro-apoptotic effect of metformin is N-cadherin & NF-kappaB dependent

**Figure 3:**
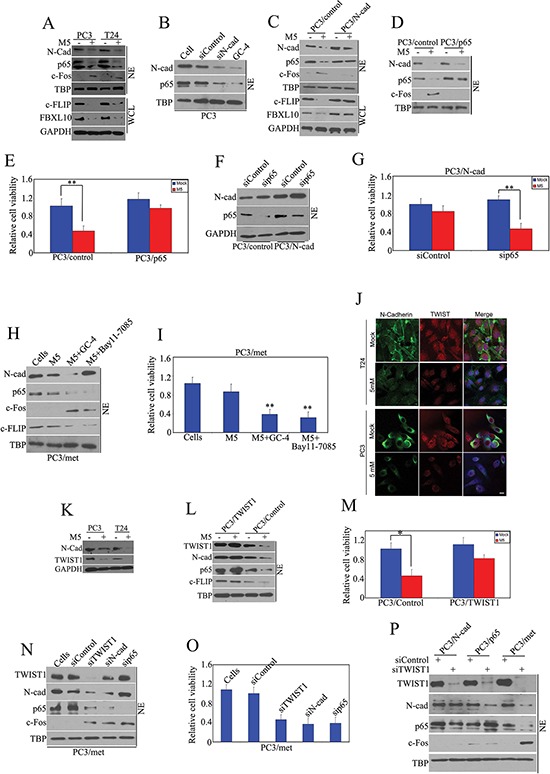
Pro-apoptotic effect of metformin is TWIST1/N-cadherin/NF-kappaB signaling dependent **A.** PC3 and T24 cells were treated with 5 mM of metformin as indicated for 48 hours and immunoblot was performed. **B.** PC3 cells were treated with N-cadherin siRNA or GC-4 (1:50) for 48 hours and N-cadherin and p65 were detected by immunoblot. **C.** Immunoblot was performed to detect protein expression in PC3/N-cad and control cells. **D.** p65 and control vector were stably transfected into PC3 cells and then immunoblot was performed to detect protein expression in the re-constructed stable cells. **E.** Cell viability was evaluated by MTS assay. **F.** p65 siRNA was transfected into PC3/N-cad stable cells and immunoblot was performed. **G.** Cell viability was evaluated by MTS assay. **H.** PC3/met cells were treated with 5 mM of metformin, combination of metformin and GC-4, combination of metformin and Bay11-7085 and vehicle. Immunoblot was performed. **I.** Cell viability was evaluated by MTS. The data represent means of average determinants ± SEM. *P < 0.05; **P < 0.01. All experiments were repeated independently at least three times with similar results. **J.** PC3 and T24 cells were treated with 5 mM of metfomin for 48 hours. Expression of N–cadherin and TWIST1 were evaluated by confocal microscopy. **K.** Expression of TWIST1, N-cadherin and p65 were detected by immunoblot. **L.** TWIST1 was stably transfected into PC3 cells and immunoblot was performed. **M.** Cell viability was evaluated by MTS assay. **N.** TWIST1, N-cadherin, p65 and control siRNA were transfected into PC3/met cells, respectively and then immunoblot was performed. **O.** Cell viability was evaluated by MTS assay. **P.** TWIST1 and control siRNA were transfected into PC3/N-cad cells, PC3/p65 cells or PC3/met, respectively and then immunoblot was performed. The data represent means of average determinants ± SEM. *P < 0.05; **P < 0.01. All experiments were repeated independently at least three times with similar results. (Scale bar, 100 μm)

NF-kappaB is a target of AKT in various tissues [14, 15], and Reiter et al have shown that increases in N-cadherin leads to up-regulation of NF-kappaB [16]. Therefore, we investigated whether the pro-apoptotic effect of metformin that is N-cadherin dependent may also be linked to NF-kappaB activity. PC3 and T24 cancer cells were treated with metformin (5 mM) for 48 hours, and then protein expression of the nuclear extract was measured by immunoblot. We found that metformin significantly down-regulated N-cadherin, p65 and its downstream molecules (c-FLIP and FBXL10 [13]), accompanied by up-regulation of c-Fos in both prostate and bladder cancer cells (Fig. 3A). Silencing of N-cadherin using siRNA and the neutralizing antibody GC-4 both downregulated expression of p65 (Fig. 3B). On the other hand, over-expression of N-cadherin concurrently upregulated p65 and its downstream molecules (c-FLIP, FBXL10) in PC3/N-cad stably transfected cells (Fig. 3C). These results suggest that metformin inhibits NF-kappaB signaling via N-cadherin, rather than directly regulating p65. We found that metformin inhibited both N-cadherin and p65 and induced apoptosis in PC3 cells (Fig. 3A–3C), but in PC3 cells with stable over-expression of p65 (PC3/p65), metformin only suppressed N-cadherin expression, but did not significantly affect expression of p65 and its downstream signaling (Fig. 3D). In addition, over-expression of p65 also compromised metformin-mediated apoptotic activities (Fig. 3E). Moreover, silencing of p65 in PC3/N-cadherin stable cells reversed the N-cadherin over-expression-mediated resistance (Fig. 3F-3G). Finally, we treated PC3 metformin-resistant cells (PC3/met) with metformin or metformin+GC-4 (N-cadherin neutralizing antibody) and metformin+ Bay11-7085 (p65 neutralizing antibody), and found that neutralizing antibody GC-4 concurrently inhibited N-cadherin and p65 and sensitized PC3/met to metformin. While, Bay 11-7085 also sensitized PC3/met to metformin, it only inhibited p65, but not N-cadherin (Fig. 3H-3I). These results demonstrate that N-cadherin/NF-kappaB signaling plays an important role in metformin-mediated pro-apoptotic activities in cancer cells that contain wild-type N-cadherin. In addition, we show that p65 is down-stream of N-cadherin, as has been shown previously [16] and suppression of p65 can overcome the effects of N-cadherin mediated resistance to metformin's anti-tumor activity.

### Metformin-mediated repression of N-cadherin is TWIST dependent

We have demonstrated that metformin induces apoptosis through N-cadherin/NF-kappaB signaling pathway. It has been reported that TWIST is a key regulator of N-cadherin [17, 18]. In order to further explore the mechanisms about how metformin regulates N-cadherin, we evaluated protein expression of TWIST1 in PC3 and T24 cancer cells after treatment with metformin and found that expression of TWIST1 was closely correlated with expression of N-cadherin (Fig. 3J–3K). TWIST was stably transfected into PC3 cells, and we found that treatment with metformin did not significantly change protein expression of TWIST1, N-cadherin and p65 (Fig. 3L) and ectopic expression of TWIST1 compromised the metformin-mediated antitumor activities (Fig. 3M). In PC3/met resistant cells, siTWIST1 inhibited TWIST1, N-cadherin and p65, accompanied by increased c-Fos. In contrast, siN-cadherin inhibited N-cadherin and p65, but did not affect TWIST1 expression, while inhibition of p65 by sip65 only inhibited p65 but not TWIST1 or N-cadherin (Fig. 3N). These results suggest that TWIST1 is upstream of N-cadherin and p65 while p65 is downstream of N-cadherin and TWIST1 (i.e.: TWIST1 —> N-cadherin —> p65 signaling pathway in metformin induced cell death). Silencing of TWIST1, N-cadherin or p65 significantly inhibited cell viability after treatment with metformin (Fig. 3O). Finally, we suppressed TWIST1 with siRNA in different stable cells and found that silencing of TWIST1 did not affect expression of N-cadherin and p65 in PC3/N-cad cells, silencing of TWIST1 did not affect expression of p65 in PC3/p65 cells, but silencing of TWIST1 downregulated TWIST1/N-cadherin/p65 signaling in PC3/met cells, and promoted apoptotic factors like c-Fos (Fig. 3P). These results showed that metformin-mediated antitumor activities may be dependent on a series of molecular signaling involving TWIST1 as an upstream mediator followed by N-cadherin and finally p65 as the most downstream mediator.

### Metformin inhibits tumor growth *in vivo*

**Figure 4:**
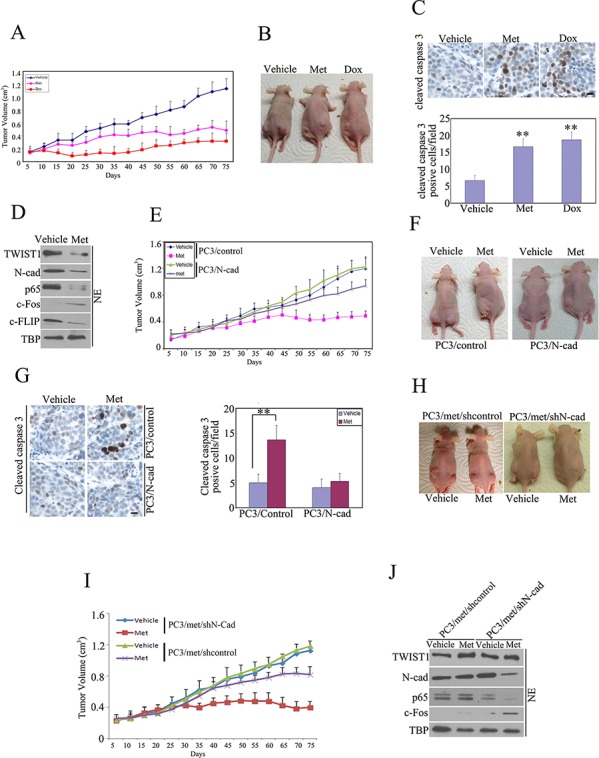
Metformin inhibited tumor growth *in vivo* PC3 cells (2 × 10^6^) were injected into both flanks of nude mice. When tumors developed to a size of approximately 0.15 cm^3^, the mice were randomly distributed into 3 groups (5 mice/group) and untreated and treated with i.p. doxorubicin (every 5 days X 4 cycles with 4 mg/kg) or p.o. metformin (200 ug/ml, diluted in the drinking water). Tumor volume was measured every 5 days. **A.** Tumor growth rates. **B.** Representative images of PC3 tumor xenografts without (−) or with (+) Metformin or Dox. **C.** Tumors were harvested at day 75 and apoptosis in the tumor was detected by measuring cleaved Caspase 3. **D.** immunoblot was performed to detect protein expression in xenografts. PC3/N-cad and PC3/control xenografts were generated and treated as described above. **E.** Tumor growth rates were measured. **F.** Representative images of tumor xenografts without (−) or with (+) Metformin. **G.** Tumors were harvested at day 75 and apoptosis in the tumor was detected by measuring cleaved Caspase 3. N-cadherin shRNA was stably transfected into PC3/met cells, and then the re-constructed cells were injected into nude mice subcutaneously to generate PC3/met/shN-cad xenografts. **H.** Representative images of tumor xenografts without (−) or with (+) Metformin. **I.** Tumor growth rates were measured. **J.** Tumors were harvested at day 75 and immunoblot was performed. The data represent means of average determinants ± SEM. *P < 0.05; **P < 0.01. All experiments were repeated independently at least three times with similar results. (Scale bar, 50 μm)

PC3 cells were injected subcutaneously into both flanks of nude mice. The PC3 xenografts that were treated with metformin resulted in decreased tumor size and higher apoptotic activities (Fig. 4A-4C) as compared to untreated group. In parallel, we found the metformin-mediated antitumor and pro-apoptotic activities were comparable to Doxorubicin-mediated antitumor activities (Fig. 4A-4C). Moreover, expression of TWIST1, N-cadehrin, p65 and c-FLIP were reduced, accompanied by increased c-Fos in metformin-treated PC3 xenografts (Fig. 4D), suggesting increased cell death activity. In contrast, over-expression of N-cadherin in PC3/N-cad xenografts demonstrated more resistance to metformin (Fig. E-G). Next, we transfected N-cadherin shRNA into PC3/met resistant cells and created stable PC3/met/shN-cad xenografts. We found that suppression of N-cadherin in resistant PC/met subline made xenografts more sensitive to metformin (Fig. 4H-4I). In addition, we found that protein expression of N-cadherin and p65 were reduced, accompanied by increased c-Fos in PC3/met/shN-cad xenografts after treatment with metformin (Fig. 4J), suggesting a more pro-apoptotic response to metformin when N-cadherin is suppressed in resistant PC3/met cells. These data further demonstrate that metformin inhibits tumor growth through TWIST1/N-cadherin/p65 pathway in in-vivo studies.

### Metformin-mediated antitumor activity in TWIST/N-cadherin deficient cells is AMPK-dependent

**Figure 5:**
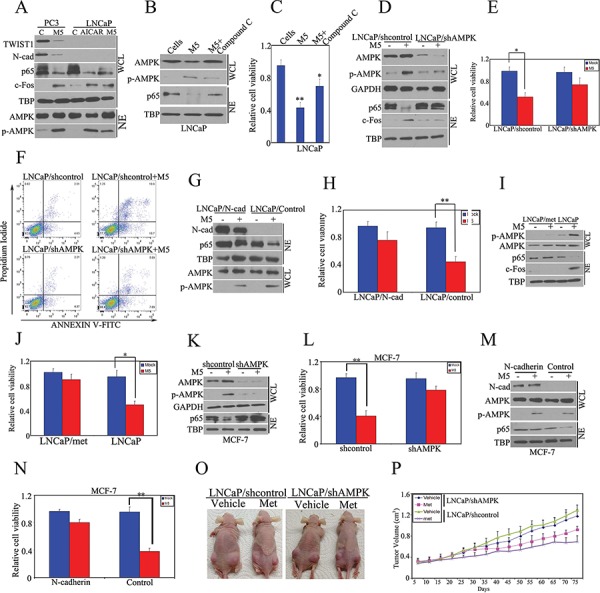
A Metformin-mediated antitumor activity in TWIST/N-cadherin deficient cells is AMPK-dependent **A.** PC3 cells were treated with metformin and LNCaP cells were treated with 5 mM of metformin, 500 μM of AICAR, respectively. Immunoblot was performed. **B.** LNCaP cells were treated with 5 mM of metformin or combination of metformin and compound C (20 μM), and immunoblot was performed. **C.** Cell viability was evaluated by MTS assay. **D.** AMPK shRNA was stably transfected into LNCaP cells and then the re-constructed LNCaP/shAMPK and control cells were treated with metformin, and immnoblot was performed. **E.** Cell viability was evaluated by MTS assay. **F.** Apoptosis was detected by Annexin V assays. **G.** N-cadherin and control vector were stably transfected into LNCaP cells and then the re-constructed LNCaP/N-cad and control stable cells were treated with 5 mM of metformin, and immunoblot was performed. **H.** Cell viability was evaluated by MTS assay. **I.** LNCaP/met cells were treated with 5 mM of metformin, and immunoblot was performed. **J.** Cell viability was evaluated by MTS assay. **K.** AMPK shRNA was stably transfected into MCF-7 breast cancer cells and then the re-constructed MCF-7/shAMPK and control cells were treated with 5 mM of metformin followed by immunoblot analysis. **L.** Cell viability was evaluated by MTS assay. **M.** N-cadherin and control vectors were stably transfected into MCF-7 cancer cells and then the re-constructed MCF-7/N-cad cells were treated with 5 mM of metformin followed by immunoblot analysis. **N.** Cell viability was evaluated by MTS assay. LNCaP/shAMPK xenografts were generated and treated as described in Figure 4. **O.** Representative images of tumor xenografts without (−) or with (+) Metformin treatment. **P.** Tumor growth rates were measured. The data represent means of average determinants ± SEM. *P < 0.05; **P < 0.01. All experiments were repeated independently at least three times with similar results.

We have demonstrated that metformin-mediated antitumor activities in wild-type TWIST/N-cadherin cancer cells require TWIST1/N-cadherin/p65 signaling and are AMPK independent. Here we wished to evaluate the metformin-induced signalling pathway in cells with absent TWIST1 and N-Cadherin expression. We used LNCaP prostate cancer cells as a model and found that there was no detectable expression of TWIST1 or N-cadherin (Fig. 5A). After exposure to metformin, expression of p-AMPK and c-Fos were increased, accompanied by reduced levels of p65. The AMPK activator, AICAR, led to similar findings (Fig. 5A). Conversely, compound C, a specific AMPK inhibitor, reversed metformin-mediated AMPK activation and down-regulation of p65, and compromised metformin-mediated inhibition of cell viability (Fig. 5B–5C). Suppression of AMPK in LNCaP cells, prevented metformin-mediated inhibition of p65 and activation of c-Fos, thus compromised metformin-mediated apoptosis (Fig. 5D–5F). Similarly, stable ectopic expression of N-cadherin in LNCaP cells (LNCaP/N-cad cells) led to persistent expression of N-cadherin and p65 despite treatment with metformin, while p-AMPK levels were elevated with metformin exposure (Fig. 5G). Over-expression of N-cadherin in LNCaP cells also compromised metformin-mediated inhibition of cell viability (Fig. 5H). In addition, we developed a LNCaP subline that is resistant to metformin (LNCaP/met) and found that protein level of p-AMPK, p65 and c-Fos were not affected after treatment with metformin, compared with parental LNCaP cells (Fig. 5I–5J), suggesting resistance to metformin and lack of metformin-induced cell death. Therefore, AMPK regulates metformin sensitivity only when TWIST1/N-cadherin signaling is deficient.

In addition to the prostate cancer cell lines, we also evaluated MCF-7 breast cancer cells, since it has been shown that TWIST1/N-cadherin signaling is deficient in MCF-7 cancer cells [19, 20]. As in LNCaP cells, metformin activated p-AMPK and reduced p65 and promoted cell death in MCF-7 cells. However, suppression of AMPK prevented metformin-mediated down-regulation of p65 and compromised metformin-mediated cell death (Fig. 5K–5L). Similarly, over-expression of N-cadherin in MCF-7 cells prevented metformin-mediated inhibition of p65 and led to resistance to metformin mediated cell death in MCF-7 cells (Fig. 5M–5N). We further confirmed our findings by xenograft experiments where AMPK was stably suppressed in LNCaP and found that inhibition of AMPK rendered the tumors more resistant to metformin. Next, we subcutaneously injected stable LNCaP/shAMPK cells into mice and generated LNCaP/shAMPK xenografts. The metformin-treated LNCaP/shAMPK xenografts resulted in decreased tumor size as compared to untreated group (Fig. O-P). Here, we have shown that in the setting of TWIST1 and N-cadherin deficiency, metformin-mediated anti-tumor activity is AMPK dependent in different cancer tissues. This finding is in contrast to cells that contain a functional TWIST and N-cadherin where metformin-mediated cell death is AMPK-independent.

### Levels of N-cadherin, NF-kB and AMPK predict post-surgical recurrence in prostate cancer after treatment of metformin

In our *in-vitro* and animal studies we have demonstrated that metformin inhibits tumor growth and apoptosis via two alternative pathways. Whether metformin inhibits TWSIT1/N-cadherin or metformin activates AMPK signaling depends on the presence of TWIST1/N-cadherin signaling. Therefore, we next asked whether these molecules could be useful clinical biomarkers of recurrence in cancer patients who are concurrently being treated with metformin for management of diabetes. To answer this question, we utilized our cohort of 984 prostate cancer patients who were surgically treated with radical prostatectomy between 1993 to 1999 and have a median follow-up of 7.9 years. To focus our attentions on individuals at risk of recurrence, we identified 49 surgically treated patients with Gleason score 7 or higher who were being treated with metformin. Among these, 26 had recurrence of prostate cancer in the form of detectable PSA or metastatic disease, while 23 patients did not have recurrence. Next, to determine whether biomarkers in the setting of metformin treatment may be predictive of prostate cancer recurrence, we evaluated the radical prostatectomy specimens from the 49 patients who were treated with metformin for TWIST1, N-cadherin, p-p65 and p-AMPK immunoreactivity (Fig. S1). We found that 16/26 patients who recurred and only 7/23 patient who did not recur with prostate cancer showed immunoreactivity to N-cadherin (*p* <0.05, Table 1). Similar findings were obtained for p-p65 (19/26 patients with immunoreactivity recurred vs. 8/23 without immunoreactivity who recurred; *p* < 0.05). To further confirm our biomarker findings, we observed the protective effect of p-AMPK expression in metformin treated prostate cancer patients where 14/23 patients with p-AMPK did not recur, whereas only 8/26 with p-AMPK expression recurred (*p* <0.05). While N-cadherin, p-p65 and p-AMPK immunoreactivity were predictive of recurrence in surgically treated prostate cancer patients who have Gleason 7 or higher disease, expression TWIST1 was not predictive of recurrence (Table 1), as a likely result of our low sample number in this select group of patients.

**Table 1:** Protein expression of N-cadherin, TWIST, p-p65 and p-AMPK by recurrence/non-recurrence group

	Recurrence (*n* = 26)	Non-recurrence (*n* = 23)
N-cad (+)	16	7
N-cad (−)	10	16
*P* value =0.04		

	Recurrence (*n* = 26)	Non-recurrence (*n* = 23)
TWIST1 (+)	16	8
TWIST1 (−)	10	15
*P* value =0.08		

	Recurrence (*n* = 26)	Non-recurrence (*n* = 23)
p-p65 (+)	19	8
p-p65 (−)	7	15
*P* value = 0.01		

	Recurrence (*n* = 26)	Non-recurrence (*n* = 23)
p-AMPK (+)	8	14
p-AMPK (−)	18	9
*P* value= 0.04		

The patient cohort findings further support our *in-vitro* and *in-vivo* studies and might explain why some patients may benefit from the anti-tumor effect of metformin. Potentially, these biomarkers may prove fruitful in predicting likelihood of sensitivity to metformin's anti tumor effects.

## DISCUSSION

Metformin is the most commonly prescribed oral anti-diabetic drug worldwide. It is used in diabetes due to excellent efficacy in reducing insulin resistance and mortality as well as fewer side effects, compared to other anti-diabetic medications [21]. In diabetic patients, metformin inhibits gluconeogenesis, stimulates glycolysis and glucose uptake into muscle and adipose tissue. Recently, the antitumor effect of metformin has been evaluated for treatment efficacy of different types of cancer [22]. Clinical and epidemiological studies have shown that metformin lowers cancer incidence and increased cumulative duration of metformin exposure in patients with cancer is associated with decreased mortality [5, 23]. Metformin may influence cancer cells indirectly by decreasing insulin levels or directly by influencing cancer cells’ viability via activation of AMPK signaling pathway [5, 7], but many other molecular mechanisms of metformin's action in cancer have also been demonstrated such as regulation of p53/REDD1 [9], modulation of DICER and c-MYC [24], and stimulation of inflammatory pathways [25]. Therefore, the molecular mechanisms of metformin's antitumor effect need further investigation. In this manuscript we have shown that molecular mechanism of metformin's antitumor activity may be largely dependent on presence or absence of N-cadherin signaling.

In cells harboring wild-type N-cadherin such as PC3 prostate cancer cells or T24 bladder cancer cells, metformin suppressed N-cadherin, and leading to decreased cellular viability. Suppression of N-cadherin by GC-4 (Fig. 1), a neutralizing antibody, or siRNA (Fig. 3O) achieved similar results. Over-expression of N-cadherin led to resistance to metformin mediated cell death (Fig. 2A–2C). In metformin-resistant PC3/met cancer cells, N-cadherin levels remained near baseline after metformin treatment in contrast to the N-cadherin levels which were reduced in the parental cell population after metformin treatment (Fig. 2J–2K), suggesting the important role that N-cadherin may have in metformin mediated cancer cell death. In in-vivo studies, we observe similar findings and show that high level of N-cadherin makes tumors more resistant to metformin and suppression of N-cadherin sensitizes tumors to metformin (Fig. 4). Ironically, in cells with wild-type N-cadherin levels, we found that AMPK expression levels did not affect sensitivity to metformin (Fig. 2D–2G). These results indicate that down-regulation of N-cadherin, rather than activation of AMPK plays a major role in metformin-mediated antitumor activities when N-cadherin is expressed. Clinical trials using N-cadherin antagonists, such as ADH-1, are currently underway and may have significant antitumor activity in different malignancies [26, 27]. Here, we have shown that N-cadherin is likely involved in mediating metformin's antitumor activity.

In order to further explore the molecular mechanisms of metformin-mediated antitumor activities, we measured NF-kappaB subunit levels, p65, a downstream molecule of N-cadherin and found that metformin regulates NF-kappaB signaling via inhibiting N-cadherin, rather than directly regulating NF-kappaB (Fig. 3A–3C). Over-expression of p65 made cancer cells more resistant to metformin (Fig. 3D–3E), while silencing of NF-kappaB sensitized cancer cells to metformin (Fig. 3F–3G). In metformin-resistant PC3/met cells, treatment with GC-4 (N-cadherin neutralizing antibody) or Bay 11–7085 (a specific NF-kappaB inhibitor) sensitized PC3/met to metformin (Fig. 3H–3I). These results suggest that metformin plays an antitumor role through N-cadherin/NF-kappaB pathway. Previously, we have shown that c-Fos [12], c-FLIP [28] and FBXL10 [13] play important roles in apoptotic mediated pathways. Therefore, we evaluated these established downstream molecules of NF-kappaB and found that treatment with metformin concurrently affects expression of c-Fos, c-FLIP and FBXL10 via NF-kappaB (Fig. 3A). Based on this result, it is very likely that c-Fos, c-FLIP and FBXL10 signaling is involved in metformin-mediated antitumor activities. Moreover, NF-kappaB plays a significant role not only in cancer, but also in inflammation, so anti-cancer effects of metformin may be also involve NF-kappaB-mediated inflammatory pathways [29].

TWIST1 and SNAIL are two important transcriptional activators of N-cadherin [18, 30, 31]. Therefore, we wished to further explore whether or not metformin could regulate N-cadherin via affecting its upstream molecules. We detected both expression of TWIST1 and SNAIL and found that metformin down-regulated N-cadherin and TWIST1 concurrently and level of N-cadherin closely correlated with level of TWIST1 (Fig. 3J–3K). However, we did not observe alterations in SNAIL levels after metformin treatment (data not shown). Ectopic expression of TWIST1 up-regulated N-cadherin/NF-kappaB signaling and led to more resistant cancer cells (Fig. 3L-3M). Suppression of TWIST1 sensitized PC3/met resistant cells to metformin (Fig. 3N–3O). In other studies, it has been demonstrated that TWIST1 also plays an essential role in metastatic cancer. Over-expression of TWIST1 is very common in malignancies. Therefore, antagonists against TWIST1 may hold further promise for cancer therapeutics [32, 33].

Because some cancer cells do not have TWIST1/N-cadherin signaling, such as prostatic cancer LNCaP cells or breast cancer MCF-7 cells, we wished to determine whether molecular mechanism of metformin's action in these N-cadherin deficiency cancer cells. We treated LNCaP cells with metformin and found that metformin activates AMPK, accompanied by reduced level of NF-kappaB signaling (Fig. 5A). Inhibition of AMPK, using a specific AMPK inhibitor, compound C or AMPK shRNA, reverses metformin-mediated inhibition of NF-kappaB and cell proliferation (Fig. 5B–5E). Over-expression of N-cadherin in deficient cells prevents metformin-mediated inhibition of NF-kappaB, and makes cells more resistant to metformin (Fig. 5G–5H). We observed similar findings in breast cancer MCF-7 cells (Fig. 5K–5N). In LNCaP/met resistant cells, treatment with metformin does not affect expression of AMPK or NF-kappaB signaling (Fig. 5I–5J). Xenograft studies showed that silencing of AMPK makes tumors more resistant to metformin (Fig. 5O–5P). Based on these results, we demonstrate that anti-cancer effects of metformin may be through activation of AMPK signaling in N-cadherin deficient cancer cells.

At last, we evaluated a cohort of 984 surgically treated prostate cancer patients with long term follow-up (median follow-up of 7.9 years). We focused on patients with Gleason grade 7 or higher cancer, since this group of patients is at highest risk of prostate cancer recurrence. We identified 49 patients who were treated with metformin during the course of their care, and found that increased expression of N-cadherin and p-p65 and reduced expression of AMPK were each associated with higher likelihood of prostate cancer recurrence (Table 1 and Fig. S1 *P* < 0.05). While additional clinical studies are required to determine the role that metformin may have in malignant processes, our initial findings suggest that biomarkers may be able to predict those individuals who may benefit the most from metformin as a drug that controls hyperglycemia and insulin resistance and as an anti-cancer agent.

**Figure 6:**
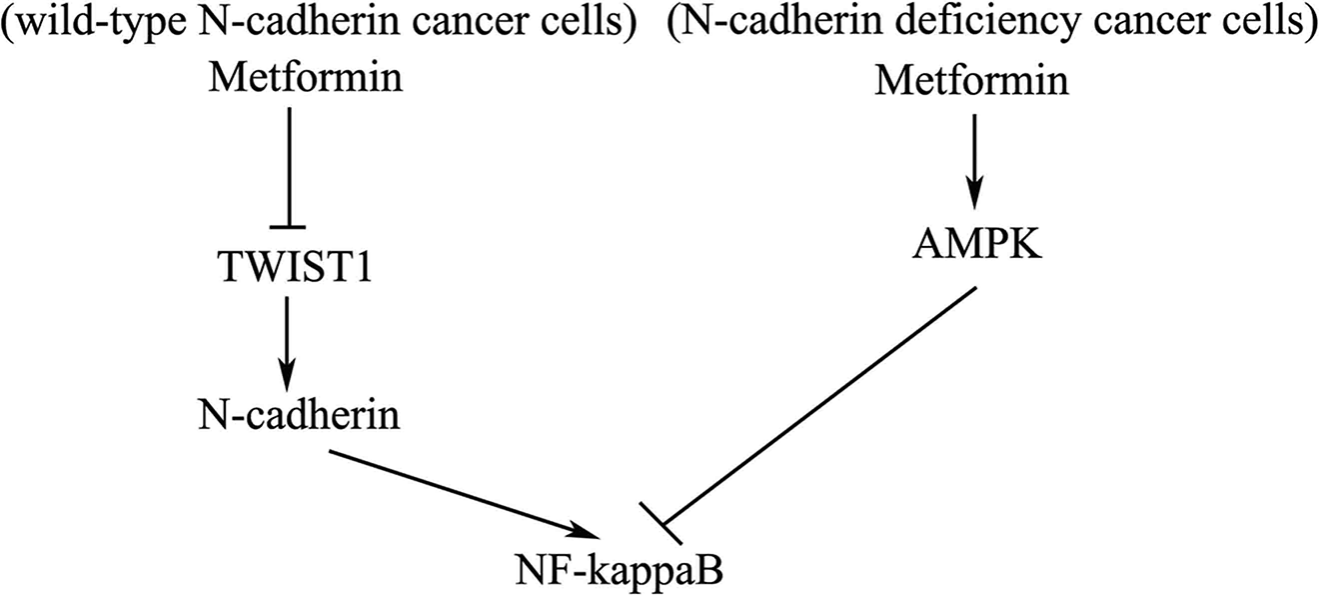
Schematic diagram of molecular mechanisms of metformin-mediated antitumor activity in N-cadherin expressing cancer cells vs. N-cadherin deficient cancer cells In N-cadherin expressing cancer cells, following metformin treatment, TWIST1 was reduced. Reduction of TWIST1 leads to down-regulation of N-cadherin, thus inhibiting NF-kappaB signaling. However, in N-cadherin deficient cancer cells, following metformin treatment, AMPK was first activated. Subsequently, activated AMPK inhibits NF-kappaB signaling.

In conclusion, we demonstrate, for the first time, that two separate pathways, AMPK-independent and AMPK-dependent pathways, may account for metformin's anti-cancer effects. We show that in the presence of N-cadherin expressing cancer cells metformin inhibits TWIST1/N-Cadherin/NF-kappaB signaling independent of AMPK (Fig. 6). In contrast, in N-cadherin deficient cells, metformin plays an antitumor role via AMPK-dependent pathways leading to subsequent inhibition of NF-kappaB (Fig. 6). These biomarkers may be useful for identification of patients who would benefit from the anti-neoplastic effects of metformin.

## MATERIALS AND METHODS

### Tissue culture, transfection

PC3, T24, 786-O, LNCaP and MCF-7 cell lines were maintained in RPMI 1640 containing 10% FCS at 37°C with 5% CO_2_. Stable PC3/N-cad, PC3/TWIST1, PC3/p65 and LNCaP/N-cad cells were established using 1 mg/ml G418 (Invitrogen) after transfection of wild-type N-cadherin, TWIST1, AMPKα1 or p65 expression vector, respectively. Transfection of plasmid and siRNA were performed using Fugene 6 or XtremeGENE according to manufacturer's instructions (Roche). PC3/met and LNCaP/met cells were metformin-resistant sublines established from parental PC3 cells or LNCaP cells by metformin treatment selection. Briefly, PC3 cells and LNCaP cells were treated with 5 mM of metformin. After 48 hours, by removing metformin and replenishing the cells with full medium, viable cells were rescued. When the plates reached 80% confluence, the cells again were treated with 5 mM of metformin for 48 hours. The cycle was repeated and PC3/met and LNCaP/met cells were generated after 10 weeks and maintained in medium with metformin.

### Reagents

Antibodies to TBP (ab51841), c-Fos (4384), GAPDH (2118), p65 (4764), N-cadherin (4061), E-cadherin (3195), AKT (4685), p-AKT (4058), Phospho-AMPKα (Thr172) (2535), AMPKα (2603), TWIST (SC-15393), FBXL10 (ab5199, ab64920), c-FLIP_L_ (06-864), cleaved Caspase 3 (9661) were purchased from Abcam, Cell Signaling and Santa Cruz. Metformin (D150959) and Doxorubicin hydrochloride (D1515), Bay11-7085 (B5681), AICAR (A9978) and Monoclonal Anti-N-Cadherin, clone GC-4 (C3865) were purchased from Sigma. shRNA AMPKα1 (TRCN0000000858), shRNA N-cadherin (TRCN0000053978), siRNA AMPK α1 (M-005027-02-0005), siRNA N-cadherin (M-011605-01-0005), Non-Targeting siRNA #1 (D-001210-01-05) and siRNA TWIST1 (M-006434-02-0005) were purchased from Dharmacon. siRNA II NF-κB p65 (6534) was from Cell Signaling. Nuclear extraction was prepared according to the manufacturer's instruction (Pierce Biotechnology).

### Cell viability and apoptosis assay

Cell viability was determined by 3-(4,5-dimethylthiazol-2-yl)-2,5-diphenyltetrazolium bromide (MTT) method according to the manufacturer's instruction (Roche Diagnostics), as previously described [28]. TUNEL assay was performed according to the manufacturer's instruction (Roche Diagnostics). The percentage of apoptotic cells was determined by analysis of Annexin V-positive cells according to manufacturer's instruction (Invitrogen). Apoptotic cells were quantified by flow cytometry (BD FACSAria I cell sorter, BD bioscience; FACSDiva™ 6.0 Software).

### Nuclear extraction, Western Blotting and immunofluorescence staining

Nuclear extraction was prepared according to the manufacturer's instruction (Pierce Biotechnology). Immunoblot was performed as previously described [28]. Metformin-treated cells were fixed in 4% paraformaldehyde, permeabilized with 0.2% Triton X-100/PBS and incubated with anti-N-cadherin, TWIST1 antibodies (1:1000), respectively, followed by incubation with Cy3-conjugated Affinipure goat anti rabbit IgG (111-167-003, Jackson Immunoresearch Lab). The sections were counterstained with DAPI and mounted with mounting medium (Vector Lab).

### Tumor Xenograft Studies

PC3 cells (2 × 10^6^) were injected into both flanks of nude mice. When tumors developed to a size of approximately 0.15 cm^3^, the mice were randomly distributed into 3 groups (5 mice/group) and untreated or treated i.p. with doxorubicin (every 5 days × 4 cycles with 4 mg/kg) or p.o. metformin (200 ug/ml, diluted in the drinking water). Tumor volume was measured every 5 days. Apoptosis in the tumor was detected by measuring cleavage of cleaved Caspase 3.

### PC3/N-cad, PC3/met/shN-cad and LNCAP/shAMPK xenografts

The stable PC3/N-cadherin and PC3/control cells were established as described previously [13]. The 1 × 10^7^ PC3/N-cad and PC3/control cells were suspended in 50% Matrigel (BD Biosciences) and injected subcutaneously into two sites per mouse (Female NCr homozygous nude mice, Taconic Farms, Germantown, NY), respectively. Tumor growth was monitored by palpation, and the onset when tumors were detectable was noted. Ten mice harboring PC3/N- cad cells and 10 mice harboring PC3/Control cells were randomly divided into two groups, respectively (each subgroup contained five mice): control groups were injected with PBS and treatment groups were treated with p.o. metformin (200 ug/ml, diluted in the drinking water). Murine body weight and tumor size were measured every five days. Tumor volume was quantified (volume = width^2^ × length × 0.52). Seventy five days after treatment, all animals were euthanized, and xenografts were harvested and assessed for immunohistochemistry and apoptosis. Tumor tissues were fixed in 10% formalin and embedded in paraffin routinely. Immunohistochemistry was carried out as previously described [34]. Tissue samples for Western blot analysis were preserved in liquid nitrogen and then prepared in radioimmunoprecipitation assay buffer with 2% SDS. The stable PC3/met/shN-cad and LNCAP/shAMPK cells were established as described previously [13]. Experimental protocol is described in “PC3/N-cad xenografts”.

### Animal Studies Ethics Permission

The mice were housed and maintained in laminar flow cabinets under specific pathogen-free conditions. All animal experiments were approved by the Institutional Animal Care and Use Committee at Massachusetts General Hospital, and performed in accordance with ethical guidelines.

### Patients

After obtaining institutional review board approval, a total of 984 patients with prostatic cancer (Gleason score ≥ 7) after radical prostatectomy (1993–1999) were included in this study. Medical records pertaining to the initial cancer diagnosis were collected, and information on cancer characteristics and treatment was extracted including tumor stage, Gleason score, type of surgery, radiation, chemotherapy and hormone therapy. Patients’ age and date of initiation of metformin therapy were collected. From our cohort of 984 patients who had undergone radical prostatectomy for management of prostate cancer, we identified 49 who were using metformin for management of diabetes and were diagnosed to have Gleason 7 or greater prostate cancer on the final surgical pathology evaluation. We evaluated the prostate cancer samples for N-cadherin, p65 and AMPK expression and assessed whether these biomarkers may have any clinical utility. Among the 49 patients, 26 patients were found to have recurrence and 23 patients had no recurrence during the followup period. Biochemical recurrence was defined as a postoperative serum PSA above the minimally detectable value. Paraffin embedded samples were used for immunohistochemical analysis.

### Statistical Analysis

Descriptive statistics were presented as mean and standard deviation. Patients with prostatic cancers were divided into 2 groups based on biochemical recurrence and non- biochemical recurrence, and descriptive analyses comparing clinical information were performed. Statistical analyses were performed with JMP Pro version 11 (SAS Institute Inc., Cary, NC). Chi-squared or Fisher's exact tests were performed for categorical variables. All tests were two-tailed, and *P* < 0.05 was considered statistically significant.

## SUPPLEMENTARY FIGURE


