# Supplementary material for: Metformin represses cancer cells via alternate pathways in N-cadherin expressing vs. N-cadherin deficient cells
Source: Oncotarget. 2015 Aug 24;6(30):28973–87. doi: 10.18632/oncotarget.5023 (PMC4745705; doi:10.18632/oncotarget.5023)
Supplement: Supplementary file 1 [file oncotarget-06-28973-s001.pdf]

SUPPLEMENTARY FIGURE

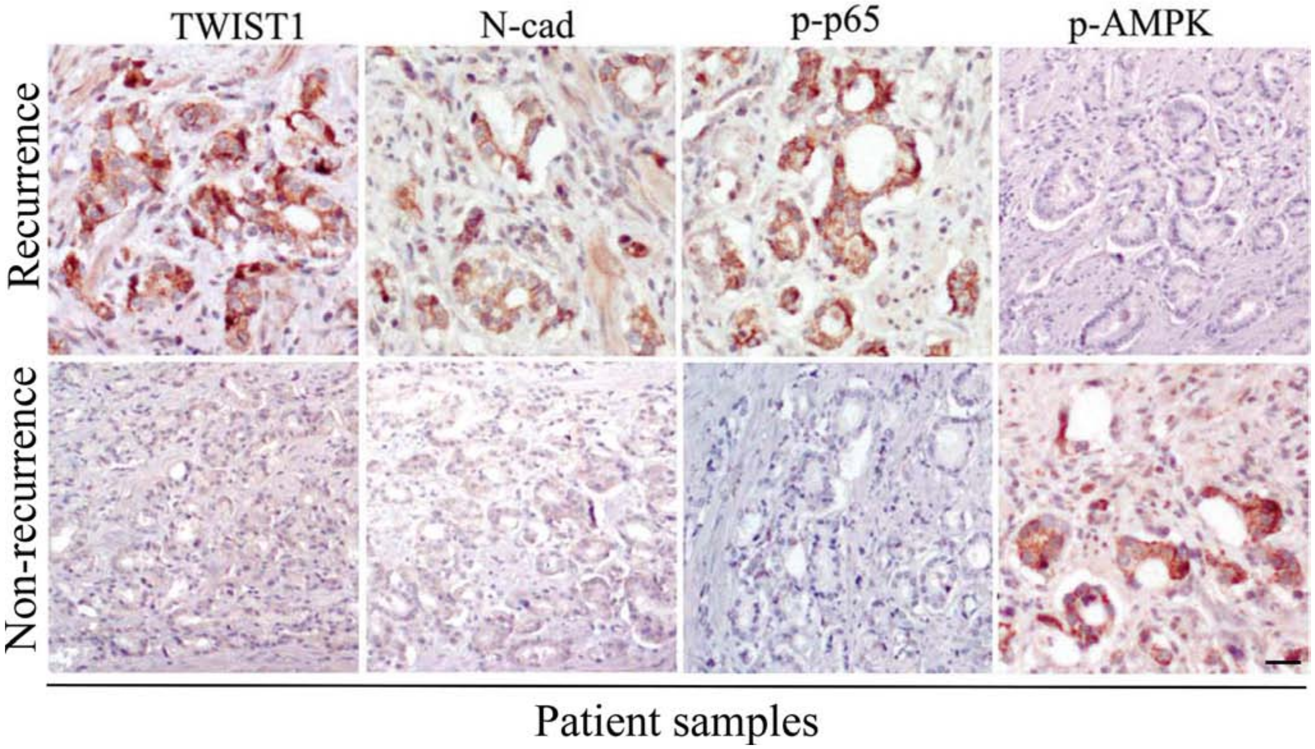

Supplementary Figure S1: Representative figures for immunohistochemical analysis of prostate cancer samples. The immunohistochemistry was defined as negative (<10%) and positive (>10%) respectively. (Scale bar, 50 µm)
